# Mesenchymal stem cell-derived exosomes: therapeutic opportunities and challenges for spinal cord injury

**DOI:** 10.1186/s13287-021-02153-8

**Published:** 2021-02-03

**Authors:** Wen-zhao Liu, Zhan-jun Ma, Jie-ru Li, Xue-wen Kang

**Affiliations:** 1 The Second Clinical Medical College, Lanzhou University, Lanzhou, 730030 Gansu China; 2 Department of Orthopedics, Lanzhou University Second Hospital, No.82 Cuiyingmen Street, Lanzhou, 730030 Gansu China; 3 School of Basic Medical Sciences, Lanzhou University, Lanzhou, 730000 Gansu China; 4 The International Cooperation Base of Gansu Province for the Pain Research in Spinal Disorders, Lanzhou, 730000 Gansu China

**Keywords:** Spinal cord injury, Mesenchymal stem cells, Exosomes, MicroRNAs

## Abstract

Spinal cord injury (SCI) often leads to serious motor and sensory dysfunction of the limbs below the injured segment. SCI not only results in physical and psychological harm to patients but can also cause a huge economic burden on their families and society. As there is no effective treatment method, the prevention, treatment, and rehabilitation of patients with SCI have become urgent problems to be solved. In recent years, mesenchymal stem cells (MSCs) have attracted more attention in the treatment of SCI. Although MSC therapy can reduce injured volume and promote axonal regeneration, its application is limited by tumorigenicity, a low survival rate, and immune rejection. Accumulating literature shows that exosomes have great potential in the treatment of SCI. In this review, we summarize the existing MSC-derived exosome studies on SCI and discuss the advantages and challenges of treating SCI based on exosomes derived from MSCs.

## Introduction

SCI is a serious neurological disease because patients often suffer from poor quality of life. In addition to motor and sensory impairment, patients also have bladder dysfunction an respiratory distress and may die [1]. According to the definition of the International Spinal Cord Society, SCI is divided into traumatic spinal cord injury and non-traumatic spinal cord injury [2]. The economic impact of SCI on patients is enormous [3].

Current treatments for SCI include surgical decompression [4–6], hemodynamic therapy [7–9], corticosteroids [10, 11], and invasive spinal cord pressure monitoring [12–14]. However, these methods do not completely restore the function of the injured spinal cord, and it is urgent to find a new method for treating SCI.

The role of mesenchymal stem cells (MSCs) in SCI has been extensively studied, but many studies have shown that MSCs have many drawbacks, and their therapeutic effects are more likely to be related to paracrine action. Exosomes are important mediators of cell-cell communication and participate in many pathological processes. The therapeutic potential of exosomes in SCI has attracted more and more attention in recent years.

This review mainly introduces the potential mechanisms of exosomes derived from MSCs in SCI. Given the unique role of exosome miRNAs derived from MSCs, we will introduce them separately. We will also discuss the prospects and challenges of MSC-derived exosomes, as MSC-exosomes may become a promising treatment method for SCI in the future.

## The pathology of SCI

The pathological process of SCI includes two consecutive processes of primary and secondary injury [15, 16]. Primary injury is defined as the immediate mechanical injury to the spinal cord, which is an irreversible process [17, 18]. Mechanical injury leads to rupture of the axonal membranes and the release of inhibitory decomposition products from the myelin sheath, such as neurite outgrowth inhibitor protein A, myelin-associated glycoprotein, oligodendrocyte myelin glycoprotein, and chondroitin sulfate proteoglycan, which are all powerful axonal regeneration inhibitors [19–25]. Physical force is the main cause of the primary injury, and this force includes forms of compression, contusion, tear, or tension [26, 27]. The secondary injury is delayed and progressive. Inflammatory cells release inflammatory cytokines due to the destruction of the blood spinal cord barrier (BSCB) [28, 29]. Secondary injury includes electrolyte abnormalities and the release of reactive oxygen species (ROS) and excitatory amino acids, which, in turn, lead to ischemia, edema, and cell necrosis, and apoptosis at the injured site [30–39]. Secondary injury is generally more complicated than the primary injury.

## Exosomes and MSC-derived exosomes

Exosomes, one of the main subclasses of extracellular vesicles that can be released into the extracellular environment, are secreted by almost all types of cells and exist widely in body fluids [40, 41]. Exosomes have clear biophysical and biochemical parameters, so they are suitable for routine laboratory tests [40, 42, 43]. The diameter of exosomes is generally 30–150 nm, and their density is 113–119 g mL^−1^ [44].

**Fig. 1:**
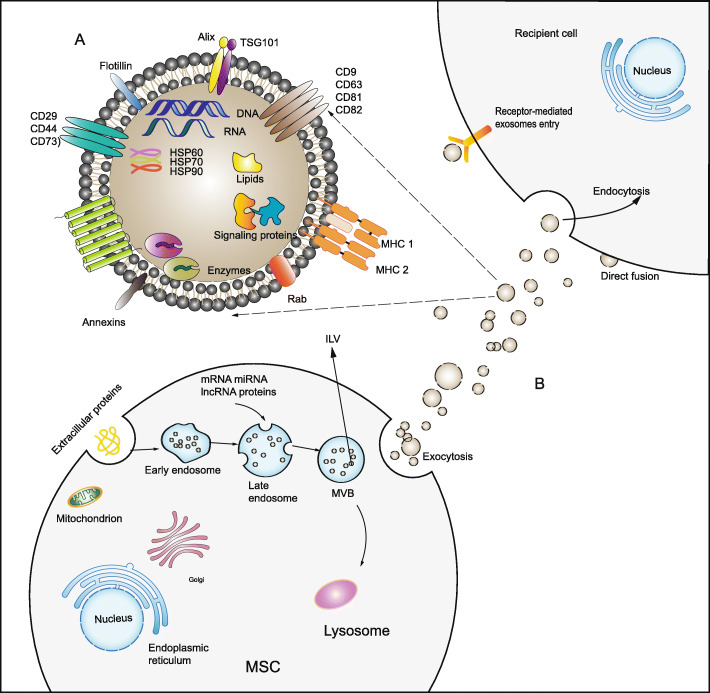
**a** The structure of exosomes derived from MSCs. MSC-derived exosomes express tetraspanins (CD81, CD63, and CD9), heat shock proteins (HSP60, HSP70, and HSP90), ALG-2 interacting protein X (Alix), TSG101, and adhesion molecules (CD29, CD44, and CD73). Exosomes derived from MSCs carry a complex cargo, including nucleic acids, proteins, lipids, and enzymes. **b** Biogenesis of MSC-derived exosomes. The biogenesis of exosomes includes the formation of early endosomes through invagination of the plasma membrane, the formation of late endosomes through selection of cargo, and the formation MVB from late endosomes. MVBs contain ILV. The fusion between MVBs and the plasma membrane results in the release of exosomes. The three ways for exosomes to enter recipient cells are receptor-mediated entry, direct membrane fusion, and endocytosis

The biogenesis of exosomes can be divided into different stages (Fig. 1), including the formation of early endosomes through invagination of the plasma membrane, the formation of late endosomes through cargo selection, and the formation of multivesicular bodies (MVBs) from late endosomes. MVBs contain intraluminal vesicles (ILVs). The fusion between MVBs and the plasma membrane results in the release of the MVB contents called exosomes [45–47]. The endosomal sorting complex required for transport (ESCRT) is an important system during exosomal biogenesis [48, 49]. However, the formation of exosomes is not entirely dependent on the ESCRT complex [50].

MSCs secrete more exosomes than other cells [51]. MSC-derived exosomes not only express tetraspanins as common exosomal surface markers (CD81, CD63, and CD9) but also express heat shock proteins (HSP60, HSP70, and HSP90), ALG-2 interacting protein X (Alix), tumor susceptibility gene 101 (Tsg101), and adhesion molecules (CD29, CD44, and CD73) [41, 52] (Fig. 1). MSC-derived exosomes, like general exosomes, carry a complex cargo, including, proteins, nucleic acids, and lipids [53, 54] (Fig. 1). In addition to cytoplasmic proteins, there are a considerable number of membrane proteins [44, 55, 56], and proteins found in lipid rafts (Flotillin-1 and Flotillin-2) [57, 58]. Exosomes are also rich in nucleic acids, which play an essential role in changing the fate of recipient cells. Among them, microRNAs (miRNAs) have been researched the most [59, 60]. miRNAs encapsulated in MSC-exosomes mainly exist in the form of their precursors [61]. Emerging evidence shows that the efficacy of MSC treatment results mainly from paracrine effects, rather than transdifferentiation and implantation of MSCs. Therefore, MSC-derived exosomes containing various paracrine mediators can be used as a cell-free therapeutic strategy [62]. And we launch the idea that MSC-exosomes have great potential to promote functional recovery and their contents may serve as biomarkers in SCI.

## Treating SCI with exosomes derived from MSCs

**Fig. 2:**
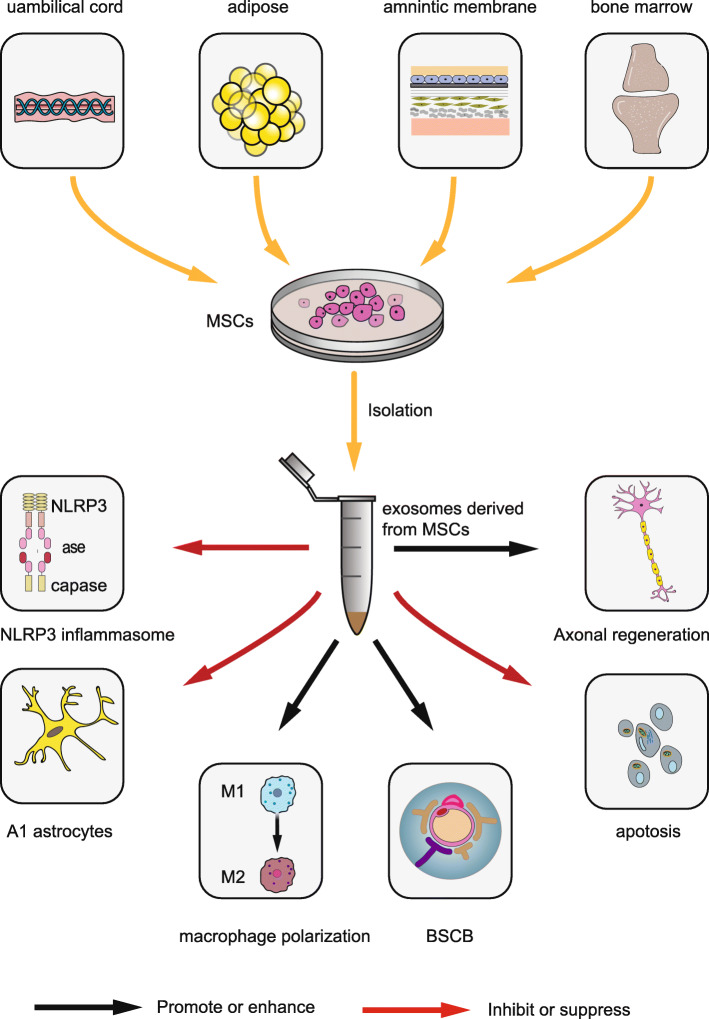
The therapeutic effects of exosomes derived from different MSCs in the treatment of SCI. MSCs can be obtained from bone marrow, the umbilical cord, the amniotic membrane, and adipose tissue. Exosomes derived from MSCs have anti-inflammatory and anti-apoptotic effects, as well as inhibit A1 astrocytes, promote axonal regeneration and macrophage polarization, and protect the BSCB from spinal cord injury

Exosomes derived from MSCs are easier to obtain and store and are subject to little ethical restriction compared with MSCs [63]. The volume of exosomes is significantly smaller than that of MSCs, so they will not be captured by lung and liver tissues, and they can penetrate the BSCB [64]. Therefore, attention has recently focused on the use of exosomes to treat SCI (Table 1). We have summarized the existing studies on MSC-derived exosomes to treat SCI. The specific mechanisms are as follows (Fig. 2).

**Table 1:** Studies on the treatment of SCI with exosomes derived from MSCs

Study	Type of MSC	Animal	Exosome diameter	Administration	Biological effects	Mechanisms of actions
Huang et al. [65]	Epidural Fat-MSCs	SD rats	60–130 nm	Intravenous injection (IV)	Alleviate cell death, attenuate tissue damage, and improve neurological recovery	Inhibit NLRP3 inflammation
Pasquale et al. [66]	hUCMSCs	SD rats	–	IV	anti-inflammatory and anti-scarring activity	Decrease the expression of pro-inflammatory cytokines and reduce astrogliosis and scarring.
Huang et al. [67]	BMSCs	SD rats	20–130 nm	IV	Attenuate cellular apoptosis and inflammation. Promote angiogenesis	Attenuate Bax expression and upregulate Bcl-2 expression. Upregulate pro-inflammatory cytokines and downregulate anti-inflammatory cytokines.
Lu et al. [68]	BMSCs	SD rats	–	IV	Attenuate neuronal cell death and Improve motor recovery	Increases BSCB pericyte coverage and decreases BSCB permeability. Inhibit pericyte migration via the NF-κB p65 pathway.
Liu et al. [69]	BMSCs	SD rats	20–150 nm	IV	Attenuate neuronal cell apoptosis and lesion size. Suppressed glial scar formation and inflammation. Promote axonal regeneration.	Suppress the activation of A1 neurotoxic reactive astrocytes. Reduce TNF-α, IL-1β, and IL-6.
Wang et al. [70]	BMSCs	SD rats	30–150 nm	IV	Reduce SCI-induced A1 astrocytes	Inhibit the nuclear translocation of NF-κB p65.
Yu et al. [71]	BMSCs	SD rats	–	IV	Accelerate the motor function and promote neuronal regeneration. Alleviate histopathological damage.	MiR-29b regulate proteins involved in neuronal regeneration, such as NF200, GAP-43, and GFAP.
Zhou et al. [72]	BMSCs	Wistar rats	40–160 nm	IV	Improve functional recovery and attenuate lesion size and apoptosis	MiR-21-5p downregulate expression of the pro-apoptotic target gene FasL.
Ji et al. [73]	BMSCs	SD rats	30–100 nm	IV	Attenuate the protective effects of obese rat MSC-derived exosomes against SCI	Insulin resistance decreased miR-21 expression in MSCs. Overexpression of miR-21 in obese rat MSCs restored the protective effects.
Xu et al. [74]	hMSCs PC12 cells	SD rats	–	IV	Suppresses the apoptosis of neuron cells and improve functional recovery	MiR-21 and miR-19b derived from the exosomes of hMSCs regulated the apoptosis and differentiation of neuron cells by regulating PTEN expression
Kang et al. [75]	–	SD rats	40–110 nm	IV	MiR-21 facilitate post-SCI recovery and suppress neuron cell death	MiR-21 inhibit the expression of PTEN/PDCD4. MiR-21/PTEN/PDCD4 signaling pathways increased cell viability and inhibited cell death in vitro
Sun et al. [76]	hUCMSCs	C57BL/6 mice	70 nm	IV	Promote locomotor functional recovery and reduce inflammation	Downregulate the inflammatory cytokines, such as TNF-α, MIP-1α, IL-6 and IFN-γ and trigger the macrophage Polarization from M1 to M2 phenotype
Li et al. [77]	BMSCs	SD rats	–	IV	Improved functional recovery, Reduced the lesion volume, Preserved neurons.	MiR-133b activate ERK1/2, STAT3, and CREB. Inhibit RhoA expression.
Li et al. [78]	BMSCs	Wistar rats	–	–	Improve locomotor functional recovery and inhibit neuronal apoptosis.	Activate the Wnt/β-catenin signaling pathway.
Zhao et al. [79]	BMSCs	Wistar rats	20–130 nm	IV	Improve functional recovery and reduce SCI-Induced complement activation.	Inhibit complement mRNA synthesis and release and inhibit activation of NF-κB signaling by binding to microglia cells.
Huang et al. [80]	BMSCs	SD rats	30–120 nm	IV	Improved functional recovery and reduced the lesion volume.	Promote angiogenesis and neurogenesis.
Yuan et al .[81]	BMSCs	SD rats	–	IV	Improved functional recovery and the axonal regeneration. Decreased the injury volume, retained the neuronal cells,	MiR-126 activates ERK1/2, STAT3 and CREB while inhibiting the expression of RhoA.
Gu et al. [82]	BMSCs	SD rats	30–150 nm	IV	Improve the recovery of motor function. Reduce neuronal apoptosis.	The expression of proapoptotic protein caspase-3 is decreased while the antiapoptotic protein Bcl-2 is upregulated, BMSC-exosomes induces activation of autophagy after SCI.

### Anti-inflammatory effects of exosomes derived from MSCs

The relative levels of pro-inflammatory cytokines, such as interleukin (IL)-1β, IL-6, and tumor necrosis factor (TNF)-α, and anti-inflammatory factors are related to the functional recovery of patients with SCI [83, 84]. Thus, the composition of the pro-inflammatory and anti-inflammatory environments is highly correlated with the prognosis after SCI, and inhibiting the formation of the pro-inflammatory environment is a major strategy for treating SCI. Romanelli et al. [66] reported that exosomes derived from human umbilical cord mesenchymal stem cells (hUCMSC-exosomes) directly interact with activated microglia in vitro and inhibit the expression of pro-inflammatory cytokines during secondary injury. Intravenous injection of hUCMSC-exosomes into an SCI rat model inhibits the expression of IL-1β and IL-6, but also inhibits the formation of scars, thereby contributing to the recovery of motor function. Neuroinflammation is characterized by the activation of resident immune cells initiated by various external stimuli, and this activation is mediated by an important protein complex-inflammasome called the nucleotide-binding domain-like receptor protein 3 (NLRP3) inflammasome that plays a key role in the secondary injury of SCI [85]. The NLRP3 inflammasome is located in the cytoplasm and is assembled by NLRP3, an apoptosis-associated speck-like protein containing a caspase recruitment domain, and caspase-1. It is involved in the regulation of the natural immune response [86, 87]. Some recent studies have shown that the activity of the NLRP3 inflammasome increases in traumatic brain injury and SCI models [85, 88, 89]. The NLRP3 inflammasome may be triggered and upregulated after SCI [88, 90, 91]. Inhibiting activation of the NLRP3 inflammasome promotes functional recovery after SCI in rats [88, 90–93]. Huang et al. [65] discovered that exosomes derived from epidural fat mesenchymal stem cells (EFMSCs) promote the recovery of neural function and reduce injured volume. The molecular mechanism is that systemic administration of EFMSC-exosomes into an SCI model significantly inhibits the activation of NLRP3 inflammasomes and reduces the expression of inflammatory cytokines. In addition, EFMSC-exosomes reduce the pro-apoptotic protein (Bcl-2-associated X protein, Bax) after SCI, while upregulating the expression of anti-apoptotic protein (B cell lymphoma-2, Bcl-2). Sun et al. [76] obtained similar results. They found that exosomes derived from hUCMSCs reduce the levels of the pro-inflammatory cytokines TNF-α, IL-6, interferon-γ, and granulocyte colony-stimulating factor while increasing the levels of the anti-inflammatory cytokines IL-4 and IL-10.

### Promotion of macrophage polarization

The therapeutic effect of MSC-exosomes has also been found to be related to the promotion of macrophage polarization. Macrophages are heterogeneous cells with extensive functional plasticity that have been divided into M1 and M2 types [94, 95]. M1 macrophages produce pro-inflammatory cytokines, ROS, and nitric oxide to promote tissue inflammation and injury. In contrast, M2 macrophages usually produce anti-inflammatory factors that reduce the ability of the injured site to produce pro-inflammatory molecules, thereby resulting in tissue remodeling. Macrophages can switch from one phenotype to another, which is induced by inflammatory factors after injury or infection [96, 97]. M1 macrophages have harmful effects in the injured spinal cord, while M2 macrophages promote axonal regeneration even in the presence of dominant inhibitory substrates [98, 99]. Most macrophages in the injured spinal cord are M1 macrophages, and only a few transient M2 macrophages exist [99]. The dominance of M1 macrophages and the decreased number of M2 macrophages after SCI aggravates the injury [98, 99]. Understanding these macrophage phenotypes and the characteristics of the chemical microenvironment after SCI will help to clarify how macrophages participate in the pathogenesis of SCI and to find new treatment strategies. Few reports are available about exosomes secreted by MSCs that promote the polarization of macrophages to treat SCI. One study reported that exosomes derived from hUCMSCs trigger the polarization of macrophages from the M1 phenotype to the M2 phenotype [76]. Lankford et al. [100] also demonstrated that intravenously injected MSC-derived exosomes quickly reach the injured spinal cord, rather than the uninjured spinal cord, and bind specifically to M2 macrophages, demonstrating that M2 macrophages can alleviate SCI.

### Reduction in A1 astrocytes

Astrocytes are very important in the process of SCI, as they can hinder or promote recovery of the CNS [101–104]. In 2017, Liddelow et al. [105] discovered that two types of reactive astrocytes, called A1 and A2 astrocytes, are induced by neuroinflammation and ischemia, respectively. A2 astrocytes play a protective role by upregulating the expression of certain neurotrophic factors, while A1 astrocytes are rapidly formed after SCI, and have neurotoxic effects on myelin, synapses, and neurons. Therefore, inhibiting A1 astrocytes is a potential treatment for SCI. A study published in 2019 confirmed that bone marrow mesenchymal stem cell (BMSC)-derived exosomes effectively promote functional recovery after SCI. One of the potential mechanisms may be to inhibit the activation of A1 neurotoxic reactive astrocytes [69]. These results suggest that applying exosome-derived MSCs may be a promising strategy for treating SCI.

A1 astrocyte marker (complement C3) will upregulate in a nuclear factor kappa B (NF-κB)-dependent manner [106]. Some studies have shown that NF-κB signaling is widely activated by a variety of pro-inflammatory agents, such as cytokines (TNF-α and IL-1) and ROS [107, 108]. In addition, the secondary inflammation of SCI is regulated by the NF-κB pathway [109], and inhibiting the NF-κB signaling pathway promotes functional recovery after SCI [110]. Wang et al. [111] reported that BMSC-derived exosome treatment effectively reduces SCI-induced A1 astrocytes by inhibiting nuclear translocation of NF-κB p65.

### Protecting the BSCB

The BSCB is responsible for maintaining the normal function of the nervous system and its unique characteristics and functions are regulated by neurovascular unit cells [112]. The BSCB is formed by the basement membrane, pericytes, capillary endothelial cells, and astrocyte foot processes [113]. Pericytes, as a part of the neurovascular unit, are very important for maintaining the integrity and barrier properties of blood vessels. Jo et al. [114] showed that the ability of pericytes to maintain the stability of microvessels mainly occurs via three possible mechanisms: promoting the expression of endothelial tight junction proteins, regulating vesicle transport and body flow across cells, and moderating the tightness connection arrangement. In neurological diseases, such as stroke and ALS, there is increasing evidence indicating that abnormal migration of pericytes aggravates these diseases [115, 116]. In clinical practice and animal models, the destruction of the BSCB is usually the inevitable result of SCI [112]. After SCI, the blood vessels at the injured site are immediately destroyed, and the BSCB far away from the injured area is permanently destroyed [117]. Therefore, maintaining the integrity of the BSCB after SCI is a potential treatment. Previous studies have shown that intravenous injection of BMSCs promotes the functional recovery of SCI in rats and accelerates the restoration of BSCB integrity [68, 118]. Further research reported that the therapeutic effect of exosomes derived from BMSCs occurs via the NF-κB p65 pathway to inhibit the migration of pericytes, thereby maintaining integrity of the BSCB after SCI, leading to a reduction of neuronal cell apoptosis, axonal regeneration, and motor function [68]. Yuan et al. [119] directly used pericyte-derived exosomes to treat SCI and found that they reduce cell apoptosis, improve microcirculation in the spinal cord after injury, and prevent BSCB injury and edema.

## Exosomal miRNAs derived from MSCs in SCI

**Fig. 3:**
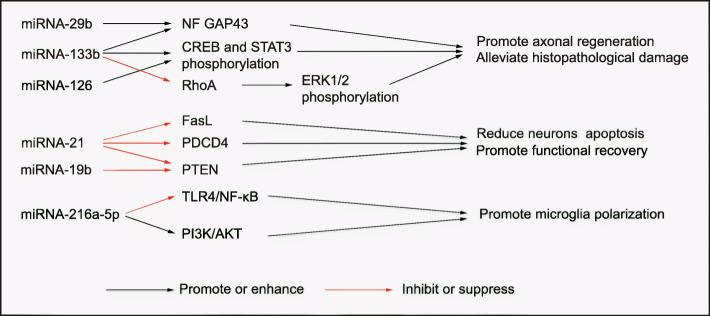
Exosomal miRNAs derived from MSCs in the treatment of SCI

MicroRNA (miRNA) is an endogenous non-coding RNA with a length of 20–24 nucleotides. After mature miRNAs are treated with dicer enzymes, they usually interact with target messenger RNAs (mRNAs) and bind to the 3′ end, leading to translational inhibition and degradation of these target mRNAs [120, 121]. Recently, some miRNAs have been identified as potential new targets for treating SCI, including miRNA-486, miRNA-21, and miRNA-126 [122–124]. Accumulating evidence reveals that exosomes with a bilayer membrane structure can be used as valuable carriers for targeting miRNAs at the SCI site. In addition, exosomes can penetrate the blood-brain barrier or BSCB to enhance the therapeutic effect of miRNAs [125]. MSCs secrete exosomes containing high levels of specific miRNAs by transfecting specific miRNA plasmids in advance [126]. Extensive studies have indicated that exosomes from MSCs carrying miRNAs have efficient repair effects on SCI. Exosomal miRNAs currently studied in SCI mainly include miRNA-21, miRNA-133b, and miRNA-126 (Fig. 3).

### MiRNA-21 of exosomes derived from MSCs in SCI

MiRNA-21 expression increases in various injured tissues and organs, suggesting that miRNA-21 is closely related to tissue injury [127–130]. Liu et al. [131] reported that miRNA-21 is unregulated in a rat model and reduces neuronal apoptosis by promoting activation of the PTEN-Akt signaling pathway and regulating the expression of the apoptosis-related proteins Bax, Bcl-2, caspase-9, and caspase-3 [132]. MiRNA-21 is one of the most common miRNAs secreted by exosomes derived from MSCs, and it is also the most studied exosomal miRNA for SCI therapeutic effects. Zhou et al. [72] determined that exosomes derived from miRNA-21-modified BMSCs significantly promote functional recovery and reduce lesion volume and apoptosis, which was mainly achieved by downregulating the expression of the pro-apoptotic gene FasL. The results of miRNA target analysis tools show that miRNA-21 contains a binding site complementary to the 3′ untranslated region of the FasL gene, indicating that the FasL gene is the direct target gene of miRNA-21. Xu et al. [74] reported that miRNA-21 of MSC-exosomes regulates apoptosis and differentiation of neurons in patients with SCI by downregulating the expression of PTEN, and that PTEN is a target gene of miRNA-21. Further research reported that miRNA-21 of exosomes derived from MSCs not only targets PTEN but also targets the tumor suppressor gene programmed cell death 4 (PDCD4). The miRNA-21/PTEN/PDCD4 signaling pathway improves cell viability and inhibits cell apoptosis [75]. Ji et al. [73] showed that the weakened protective effect of exosomes derived from MSCs on SCI in obese rats was due to insulin resistance in the rats. Insulin resistance of MSCs reduces the level of miRNA-21 secreted by exosomes, which further strengthens the view that miRNA-21 is a potential molecule for treating SCI.

### MiRNA-133b of exosomes derived from MSCs in SCI

MiRNA-133b plays an important role in neuronal differentiation, growth, and apoptosis [133–135]. Some studies have shown that overexpression of miRNA-133b promotes functional recovery after stroke in rats [136, 137]. In addition, studies on zebrafish and rodents have indicated that miRNA-133b is expressed in midbrain dopaminergic neurons where it regulates the production of tyrosine hydroxylase and dopamine transporters in patients with Parkinson’s disease [138]. In a study on the relationship between miRNA-133b and functional recovery after SCI in adult zebrafish, Yu et al. [139] showed that decreased expression of miRNA-133b is not conducive to the recovery of motor function and reduces neuronal axonal regeneration after using morpholino antisense oligonucleotides, which inhibit the expression of miRNA-133b.

Some molecules mediate the protective effect of miRNA-33b on SCI, such as signal transducer and activator of transcription 3 (STAT3), RhoA, and cAMP-response element-binding protein (CREB). STAT3 is distributed in astrocytes and neurons and is responsible for neuronal proliferation and differentiation as well as axonal regeneration [140, 141]. Activated STAT3 mediates inflammation caused by SCI [142]. RhoA is a member of the Rho family that is upregulated after SCI in rats and acts on Rho-associated kinase [143], which is its direct downstream effector. RhoA is related to the death of neurons [144]. The transcription factor CREB also plays an important role in axonal regeneration [145]. Activation of CREB is sufficient to overcome myelin inhibitors and promote axonal regeneration in vivo [146]. Qi et al. [147] demonstrated that miRNA-133b in exosomes significantly increases the STAT3 phosphorylation level, which is involved in axonal regeneration in the injured spinal cord of SCI rats. There is evidence that RhoA is a direct target of miRNA-133b [134]. In addition, miRNA-133b in exosomes released by MSCs promotes axonal growth [148]. Li et al. [77] further demonstrated this result and showed that systemic injection of miRNA-133b exosomes protects neurons and promotes the recovery of motor function after SCI, and this effect was at least partially due to activation of ERK1/2, STAT3, and CREB and inhibition of RhoA expression. Ren et al. [149] showed that exosomes containing miRNA-133b significantly promote the expression of neurofilament, growth-associated protein 43 (GAP-43), glial fibrillary acidic protein, and myelin basic protein by affecting signaling pathways related to axonal regeneration and promoting recovery of neuronal function in SCI animals.

### MiRNA-126 of exosomes derived from MSCs in SCI

MiRNA-126 has recently been found to promote functional recovery after SCI. In 2015, Hu et al. [124] reported that the expression of miRNA-126 decreases after SCI, while increasing the level of miRNA-126 reduces inflammation and promotes angiogenesis and functional recovery. This process may be related to the downregulation of sprouty-related EVH1 domain-containing protein 1, phosphoinositol-3 kinase regulatory subunit 2, and vascular cell adhesion molecule 1 target gene expression. Some scholars have turned their attention to using exosomes derived from miRNA-126-modified MSCs to treat SCI. Huang et al. [80] demonstrated that exosomes containing miRNA-126 promotes angiogenesis and neurogenesis after SCI, as well as attenuates cell apoptosis, thereby promoting functional recovery of an SCI rat model. Yuan et al. [81] indicated that systemic administration of exosomes derived from miRNA-126-modified MSCs promotes functional recovery and axonal regeneration. Similar to miRNA-21, it is likely that miRNA-126 activates ERK1/2, STAT3, and CREB while inhibiting the expression of RhoA.

### Other miRNAs of exosomes derived from MSCs in SCI

In 2019, Yu et al. [71] injected exosomes secreted from miRNA-29b-modified BMSCs into a rat model. They showed that these exosomes accelerate motor functional recovery and reduce pathological damage of spinal cord tissue in rats with SCI, as well as promote neuronal regeneration. This mechanism may also be related to regulating the expression of neural regeneration-related proteins, such as NF200, GAP-43, and GFAP. In addition, Zhao et al. [150] found that exosomes derived from BMSCs have neuroprotective effects in the ischemic spinal cord. This effect may be due to pre-transfection of BMSCs to secrete exosomes with high expression of miRNA-25, thus indicating that miRNA-25 enhances neuroprotection. Another exosomal miRNA that has neuroprotective effects is miRNA-544. Li et al. [151] transfected rat BMSCs with miRNA-544 mimic to obtain exosomes that highly expressed miRNA-544 and these exosomes were intravenously injected into a SCI rat model. The results showed that miRNA-544 accelerates the recovery of neuronal function after SCI. In addition, overexpression of miRNA-544 in BMSC-exosomes alleviated the histological defects and neuronal loss caused by SCI. Liu et al. [152] determined that exosomal miRNA-216a-5p transforms microglia from the M1 pro-inflammatory phenotype to the M2 anti-inflammatory phenotype by inhibiting TLR4/NF-κB and activating the PI3K/Akt signaling pathway, thereby increasing treatment potential.

## Challenges and prospects

The main obstacles to the repair of an injured spinal cord include the weakened ability of axonal growth, insufficient repair of endogenous cells, and the presence of inhibitory molecules at the injured site [153–156]. Overcoming these obstacles would lead to an ideal method for treating SCI. MSC transplantation seems to be an attractive option. However, the direct transplantation of MSCs has potential risks. For example, one study reported that BMSCs that have not been genetically modified could have chromosomal abnormalities even during early passages, leading to the formation of malignant tumors [157]. Moreover, MSCs cannot differentiate into neurons. Immunochemistry, molecular marker, and cell morphology studies indicate that although MSCs have neuron-like characteristics after transplantation, it is difficult to regard them as real neurons [158, 159]. The expression of neuronal antigens may simply be due to the immature nature of the MSCs [160]. During in vitro culture, MSCs gradually lose their potential to proliferate and differentiate [161, 162]. According to current evidence, the curative effect of MSCs seems to be related to their paracrine activity but has little to do with the mechanism of cell replacement [163]. Moreover, the disadvantages of MSCs, such as tumorigenesis, low survival rate, and immune rejection, make it difficult to continue the treatment of SCI using MSCs [164].

Similar to MSCs, MSC-exosomes have the same characteristics of homing to the injured tissue, and have the advantage of nanometer size, allowing them to pass through the BSCB and play an important role in the repair of the nervous system. More importantly, based on their relatively small molecular structure, natural molecular transport characteristics, and good biocompatibility, exosomes have shown great application potential as drug carriers in recent years. Traditional drugs often have a number of defects, such as poor water solubility, quick removal by the body, poor biocompatibility, unsatisfactory distribution in vivo, and low permeability to cells, which limit their efficacy and clinical application. However, exosomes combine the advantages of cell and nanotechnology in drug delivery. For example, exosomes improve the stability of drugs; exosomes have a natural targeting ability based on donor cells when delivering drugs, and exosomes are nano-molecules with cell surface substances, so they have strong biological barrier permeability and can selectively penetrate a tissue injury. Therefore, we speculate that exosomes will be a promising drug-delivery system to treat SCI. Another advantage of exosomes derived from MSCs is that they are not tumorigenic. No study has reported on the tumorigenesis potential of MSC-exosomes [165]. Although there is a lack of a direct comparison of the characteristics of exosomes derived from different MSCs in SCI models, we believe that umbilical cord mesenchymal stem cells (UCMSCs) may be one of the best sources because they are easier to obtain than BMSCs, and do not involve ethical issues.

Although exosomes have great potential to treat SCI, there are still a number of challenges that need to be addressed before exosome therapy for SCI can be used in clinical trials. First, the source of the exosomes must be determined, and the content and function of exosomes obtained under different culture conditions (such as hypoxia and growth factors) are also not consistent [166]. In addition, MSC-exosome separation methods must be standardized. There is no consensus on the method to separate exosomes, and different methods of separating exosomes have advantages and disadvantages. In fact, there are significant differences in protein and RNA contents among different separating methods [167]. The most commonly used method is ultracentrifugation, but the purity of exosomes obtained by this method is low, and the exosomes can be contaminated by other EVs with similar diameters [168], so it is necessary to explore a more efficient method. Another problem that needs to be solved is the storage, preservation, and transportation of exosomes. Although exosomes are more stable and suitable for long-term preservation than MSCs, a study published in 2018 showed that it is possible to purify exosomes by lyophilization [169]. This would help produce ready-to-use batches of exosomes, which could be easily transported; however, further research is needed to demonstrate whether lyophilization will change the characteristics of exosomes. Before exosomes can be used in clinical trials it will also be necessary to verify the half-life of freshly isolated and cryopreserved exosomes after injection. The contents of exosomes also need to be further studied to understand which components can be used to treat SCI and which may be harmful. Further research is needed to probe the relationship between injection frequency, dosage, and the therapeutic effect of MSC-exosomes to maintain the long-term effect, and whether single or multiple administrations will have a negative effect, which is very important for the correct use of exosomes to treat SCI. Research on the treatment of SCI with exosomes derived from MSCs is in the exploratory stage; the number of studies is small and most of them are based on rodents, particularly Sprague-Dawley rats. However, there are anatomical differences between human and rodent spinal cords. The SCI area of the rodent model is small, while the SCI area of humans is often larger, which leads to more tissue loss. Additionally, the human nervous system is more complex and more advanced than that of rodents. The process of human SCI is characterized by an immune response, a vascular response, an inflammatory reaction, and glial scar formation, which is also significantly different from SCI in rodents. Thus, the scope of research needs to be expanded further using larger animals (such as dogs) for research. In addition, although exosomes have a demonstrated therapeutic effect on SCI, the specific therapeutic mechanism and target are not exactly clear, and most studies have focused on the role of miRNAs; there is less research on the role of other components of exosomes, so further research is needed to clarify the therapeutic effects of exosomes. Furthermore, although MSC-exosomes are superior to MSCs in the treatment of SCI, the production technology for MSC-exosomes needs to be improved before it can be used in clinical practice. Studies have shown that MSCs secrete only a small number of exosomes (1–4 μg of exosome protein can be extracted from 10^6^ cells/day) [170]. Therefore, long-term cell culture and a large number of MSCs are needed to produce sufficient numbers of exosomes for clinical applications. However, the expression of growth factors decreases significantly in late-passage MSCs, which would reduce the therapeutic effect of growth factors and its mRNAs secreted by exosomes [171]. As mentioned earlier, the obstacles to SCI recovery include the weakened ability of axonal growth and insufficient endogenous cell repair but, unfortunately, current research on MSC-derived exosomes does not aid recovery through these mechanisms. In addition, there is a lack of research for horizontal comparison of exosomes from different MSCs in SCI, and the differences in the therapeutic efficacy of exosomes from different MSCs remain unclear.

## Conclusions

In conclusion, the treatment of SCI is a great challenge, and there is no effective strategy to restore lost function. The pathological process of SCI is very complex and is the result of multiple factors, which hinders the development of treatments leading to a full recovery. Therefore, understanding the pathological mechanism is conducive to better treatments for SCI. Because of the poor plasticity and weak regenerating ability of the CNS, the recovery of neural function is greatly limited. As an intercellular communication medium, exosomes are superior for treating SCI, particularly exosomes derived from MSCs. Exosomes derived from MSCs can pass through the BSCB and can be used as good drug carriers, which has great therapeutic potential in SCI. We must optimize MSC-derived exosomes to improve their therapeutic effect in SCI. More research is required to clarify the specific role of exosomes in SCI. If these problems can be solved, it will provide a comprehensive theoretical basis for the clinical transformation of MSC-derived exosomes in the treatment of SCI, and bring hope for clinical treatment of SCI.

## Data Availability

Not applicable.

## Abbreviations

Alix: ALG-2 interacting protein X
Bax: Bcl-2-associated X protein
BBB: Blood-brain barrier
Bcl-2: B cell lymphoma-2
BMSCs: bone marrow mesenchymal stem cells
BSCB: Blood spinal cord barrier
CM: Conditioned medium
CNS: Central nervous system
CREB: cAMP-response element-binding protein
EFMSCs: Epidural fat mesenchymal stem cells
ESCRT: Endosomal sorting complex required for transport
GAP-43: Growth-associated protein 43
GFAP: Glial fibrillary acidic protein
hUCMSCs: Human umbilical cord mesenchymal
IV: Intravenous injection
hMSCs: Human mesenchymal stem cells
HSP: Heat shock proteins
IFN-γ: Interferon-γ
ILVs: Intraluminal vesicles
miRNAs: MicroRNAs
MSCs: Mesenchymal stem cells
MVs: Microvesicles
MVBs: Multivesicular bodies
NF: Neurofilament
NF-κB: Nuclear factor kappa B
NLRP3: Nucleotide-binding domain-like receptor protein 3
PD: Parkinson’s disease
PDCD4: Programmed cell death 4
PTEN: Phosphate and tension homology deleted on chromsome ten
ROS: Reactive oxygen species
SCI: Spinal cord injury
STAT3: Signal transducer and activator of transcription 3
TLR4: Toll-like receptor 4
TNF-α: Tumor necrosis factor-α
Tsg101: Tumor susceptibility gene 101
UCMSCs: Umbilical cord mesenchymal stem cells
